# Mental Health and Loneliness in University Students During the COVID-19 Pandemic in Germany: A Longitudinal Study

**DOI:** 10.3389/fpsyt.2022.848645

**Published:** 2022-04-15

**Authors:** Maxi Weber, Lars Schulze, Teresa Bolzenkötter, Helen Niemeyer, Babette Renneberg

**Affiliations:** ^1^Clinical Psychology and Psychotherapy, Freie Universität Berlin, Berlin, Germany; ^2^Clinical Psychological Intervention, Freie Universität Berlin, Berlin, Germany

**Keywords:** COVID-19, pandemic, depression, anxiety, loneliness, students, university

## Abstract

The COVID-19 pandemic and its preventive measures had adverse consequences for mental health. However, knowledge of mental health trajectories across the pandemic is limited. This study investigated the mental health levels and changes among university students during the pandemic and lockdown in Germany, as well as their associated factors. We surveyed students' mental health (*N* = 363, 68% female) with the patient health questionnaire (PHQ-8) and the generalized anxiety disorder scale (GAD-7) during the first easing phase (July 2020; time 1) and the second lockdown (November 2020; time 2). Cut-off scores from the GAD-7 and PHQ-8 were used to determine clinically relevant symptoms and to define trajectory groups. Sociodemographic and pandemic-related data were assessed (e.g., coping with academic life, social contacts) as well as loneliness, stress, repetitive negative thinking, quality of life, and perceived social support. Paired *t*-test, multiple regression, and repeated-measures ANOVA were applied. Means and prevalence rates for symptoms of depression (38.8%) and anxiety (25.6%) did not differ between time 1 and time 2, and most students were asymptomatic on the PHQ-8 (44.4%) and the GAD-7 (56.3%) across the pandemic. Feelings of loneliness significantly increased from time 1 to time 2, *d* = −0.30, [−0.47, −0.13], with higher symptom levels in symptomatic groups at time 2 and greater increases in the asymptomatic groups. Levels of stress, repetitive negative thinking, quality of life, and social support did not differ during the pandemic. At time 1, loneliness and repetitive negative thinking were associated with anxiety and depressive symptoms. Anxiety and depressive symptoms were prevalent among students, and increased levels of loneliness during the pandemic were associated with elevated symptoms and differing trajectories. Further research using representative and larger samples should determine the long-term impact of the pandemic on mental health and loneliness to identify vulnerable students and offer adequate support.

## Introduction

The first wave of the coronavirus disease (COVID-19) pandemic and its lockdown measures negatively affected the mental health of many individuals (1, 2). However, specific subgroups at higher risk for mental health problems were described, including university and college students (3–7). Even before COVID-19, students were exposed to multiple stressors during the emergent adulthood adapting to social and academic life (8–11). With the COVID-19 pandemic, further potential stressors emerged due to closed universities, remote learning formats, and prolonged social distancing measures.

Studies across the globe revealed elevated symptoms of anxiety, depression, stress, and loneliness among university and college students during the pandemic (12–23). However, most of the studies applied cross-sectional designs and mental health impacts should be interpreted with caution (24). Some studies provided longitudinal data comparing the same students before and closely after the first peak of the pandemic to examine how mental health has changed. Compared with pre-pandemic levels, the majority of studies likewise showed increased symptoms of depression, anxiety, and stress (12, 25–32), but not all (33). Mixed findings were reported by Meda et al. (34) showing increased symptoms for depression but not for anxiety among students during the first lockdown in Italy compared to pre-pandemic levels, while another study among medical students in India suggested increased rates for anxiety but not for depression (35).

A few additional longitudinal studies compared different time points during the pandemic to examine how mental health has changed and revealed conflicting results. For example, studies among Chinese college students found increased anxiety and depression rates when the pandemic was under control compared with the acute phase of the pandemic, but not for self-reported stress (22, 36). Two other studies indicated decreased anxiety and depression symptoms (37), and stress levels (38) during the first lockdown compared to the pre-pandemic academic semester before increasing again in the post-lockdown period. In contrast, others reported generally high but declining anxiety and depression symptoms along with reduced daily COVID-cases and eased lockdown measures in Italy (34) and the United States (39). One recent repeated cross-sectional study with a large sample of students and non-students compared anxiety and depressive symptoms at three pandemic time points in France (40). Relative to non-students, students showed higher depressive symptoms during the first national lockdown (19% vs. 36%), comparable rates during the easing phase (21% vs. 27%), and again dramatic increases during the second lockdown (27% vs. 54%). Symptoms of anxiety were likewise more prevalent in students compared with non-students during the pandemic.

In addition to the mixed findings on mental health courses during the pandemic, it is less understood which risk and protective factors co-determine mental health levels and changes among university students during the pandemic. Previous cross-sectional data largely based on the general population suggested that increased symptoms of anxiety and depression were associated with female gender, younger age, living alone, and financial insecurities (3, 23, 41–47). In addition, these studies implied that adverse coping styles, repetitive negative thinking, boredom, pre-existing mental health conditions, and adverse childhood experiences were associated with worsened mental health, while perceived social support, having social contacts, and self-efficacy were protective for mental health.

In recent years, loneliness has consistently been linked to poorer mental health, symptoms of anxiety and depression. (e.g., (48–50). With the COVID-19 pandemic and the established social distancing measures, the link between loneliness and mental health was further emphasized (4). In fact, loneliness during the pandemic increased in the general population (51) as well as in university students compared with pre-pandemic levels (12, 29), and this increase was more prevalent in students compared with non-students (52). Moreover, loneliness was largely responsible for the exacerbated course of depressive symptoms in young adults during the pandemic (53).

Overall, knowledge is limited regarding the mental health levels of university students and trajectories after prolonged threats and stressors during the COVID-19 pandemic. Longitudinal research is warranted on how mental health changes and which stressors may be of primary concern to target prevention efforts, particularly following multiple lockdowns. In Germany, the first lockdown started in March 2020 with easing steps from May to June 2020; the second four months later in November 2020 to May 2021. Most shops, restaurants, and universities were closed and gatherings of more than five people were banned. Between the two lockdowns, restaurants and shops re-opened and contact restrictions were eased while universities remained closed. Additional measures were maintained during the easing phase such as a minimum distance of 1.5 m to others, wearing face masks in public transport, and the recommendation to reduce physical contacts whenever possible.

Using a cross-sectional and longitudinal cohort design with measures during the easing phase and the second lockdown in Germany, the current study aimed at 1) investigating the levels and changes of mental health in university students, and 2) identifying associated factors for mental health (i.e., sociodemographic, pandemic-related, and psychological variables). As registered previously (osf.io/na5b6), we expected overall worsened primary anxiety and depressive symptoms in students and worsened psychological outcomes (i.e., loneliness, stress, repetitive negative thinking, quality of life, social support) along with increasing COVID-19 cases and deaths, and re-introduced lockdown measures. To better understand how mental health changed during the pandemic, we examined trajectories based on the clinical cut-off scores for probable anxiety and depression at each time point and their associations with changes in loneliness. Moreover, we tested whether anxiety and depressive symptom levels were associated with sociodemographic variables (e.g., female gender, living alone, socioeconomic status), pandemic-related variables (e.g., coping with daily and academic life, reduced social contacts since the pandemic), and psychological variables (e.g., higher levels of loneliness, ruminative thinking, lower perceived social support, current mental disorder).

## Methods

### Study Design and Participants

This cross-sectional and longitudinal online survey study was conducted according to the Strengthening the Reporting of Observational Studies in Epidemiology guidelines for observational studies (54). Data was collected 2 months after the first lockdown in Germany (20 July−28 August 2020; time 1), and during the second lockdown when rates of COVID-19 infections and related deaths increased dramatically (10 November−2 December 2020; time 2). Participants older than 18 years studying in Berlin, Germany (no other inclusion or exclusion criteria) were recruited via social media, mailing lists, and the institutional website. The study was approved by the ethics committee at Freie Universität Berlin (032/2020). *N* = 467 students provided informed consent and initiated the survey via the *Questback* platform. We included *n* = 363 (77.7%) participants for the cross-sectional analyses at time 1 with complete questions regarding the primary outcomes, of which 343 (94%) completed the whole survey. *N* = 254 participants agreed to participate in the survey at time 2, of which 157 respondents completed the whole survey. Matched data at time 1 and time 2 was available for 135 respondents. This sample size entailed more than 90% power to observe a small within-effect at the 5% level (G^*^Power 3.1.9.2, *F*-test, repeated-measures ANOVA). Participants completing the survey at time 1 were entered into a raffle to receive one of ten 25 € gift cards. Psychology students from Freie Universität Berlin could receive course credits after each wave.

### Measurements

#### Sociodemographic and Pandemic-Related Variables

The questionnaire battery at time 1 comprised data related to age, gender, family status, living situation, highest degree, field of study, students' income, and the socioeconomic status indexed by the degree and profession of the student parents (55).

To measure pandemic-specific experiences, additional items were formulated. Participants rated their perceived wellbeing and their coping abilities in daily life, in academic life, and with a potential future lockdown on a five-point Likert scale, ranging from 1 “good” to 5 “poor” (e.g., *How have you been feeling in general since the pandemic?*). Perceived wellbeing and finances compared to before the pandemic were rated on a five-point Likert scale, ranging from 1 “strongly improved” to 5 “strongly worsened” (e.g., *Has your financial situation changed since the pandemic?)*. Finally, participants reported the number of days during the last 2 weeks (0–14 days) that they had social contacts and consumed alcohol, respectively (see osf.io/na5b6 for study materials used).

#### Primary Mental Health Outcomes

Anxiety and depression symptoms were assessed with the well-validated 7-item Generalized Anxiety Disorder Scale [GAD-7, (56, 57)] and the 8-item Patient Health Questionnaire [PHQ-8, (58)] with equivalent diagnostic accuracy compared to the PHQ-9 (59). Total scores range from 0 to 21 for the GAD-7 and from 0 to 24 for the PHQ-8. Scoring 10 or above indicates moderate-to-severe symptomatology, which typically represents clinically significant depression and anxiety (58, 60). Both instruments demonstrated excellent internal consistency in the present study (GAD-7, ɑ = 0.87; PHQ-8, Cronbach's ɑ = 0.86). Open-ended responses toward the most distressing and most positive experiences and perceived changes in academic life during the last 2 weeks were gathered to cross-validate symptoms levels, which will be presented elsewhere in detail.

#### Additional Psychological Variables

Outcomes assessed with reliable and valid questionnaires at the two time points were loneliness [UCLA loneliness scale, ULS-8, (61)] stress [perceived stress scale, PSS-10, items 3, 6, (62, 63)], quality of life [satisfaction with life scale, SWLS, (64, 65)], repetitive negative thinking [perseverative thinking questionnaire, (66)], and social support [brief form of perceived social support questionnaire, (67)]. Single-measure items were applied to measure feelings of boredom, presence of diagnosed mental disorder, and subjective health at time 1 (68). Associated factors with mental health at time 1 included coping strategies [active coping, positive reframing, acceptance, religion, and substance use, Brief-COPE, (69, 70)], self-efficacy [generalized self-efficacy scale; (71)], social anxiety [mini social phobia inventory, (72)], and adverse childhood experiences [ACE, (73)]. The applied scales proved acceptable to excellent internal consistency in this study (Cronbach's ɑ = 0.72–0.96), except for the coping subscales religion and active coping (ɑ = 0.64, 0.69), which were subsequently removed from further analyses.

#### Data Analysis

The Welch's *t*-test, univariate analysis of variance (ANOVA) and Chi-square test of independence were used to test differences between the cross-sectional sample and the longitudinal sample. To determine research question 1) regarding the mental health levels and changes during the pandemic, paired sample *t*-tests and their respective effect size estimates using Cohen's *d* were used to examine mean changes in variables tested at time 1 and time 2 (i.e., anxiety, depression, loneliness, stress, quality of life, social support, and repetitive negative thinking). Cohen's *d* of 0.2, 0.5, and 0.8 indicate a small, medium, and large effect size, respectively (74). Regarding the dependent variables anxiety and depressive symptoms, clinically relevant symptom levels at time 1 and time 2 were determined using the established cut-off score of 10 of the GAD-7 and PHQ-8 (58, 60). To adjust trajectories during the pandemic [e.g., (75)], the cut-off scores at time 1 and time 2 were used, resulting in four potential paths for anxiety and depression: 1) the asymptomatic, 2) the worsened, 3) the symptomatic, and 4) the improved trajectory. To further explore how mental health changed during the pandemic, two-way repeated measures ANOVAs with loneliness as the dependent variable were performed with the factors time and trajectory groups for depression and anxiety. Post hoc analysis using the Tukey's test (76) were applied as well as partial eta squared $\left(\eta_{p}^{2}\right)$ as measures of small (0.01), medium (0.06), and large (0.14) effect sizes (74).

To examine research question 2) on factors associated with increased anxiety and depressive symptoms, we performed multiple linear regression analyses for the two primary outcomes individually, as others have done (15, 77). The two models tested associations measured at time 1, respectively, with sociodemographic (e.g., age, gender, living situation, socioeconomic status), pandemic-related (e.g., coping with academic life since the pandemic, social contacts), and additional psychological variables (e.g., loneliness, social support, presence of current mental disorder). Associated factors for the two primary outcomes were first examined in univariate linear regression analyses and subsequently entered into a multiple linear regression model adjusting for all other tested sociodemographic, pandemic-related, and psychological factors. The statistical assumptions were tested regarding multicollinearity (i.e., tolerance and VIF factor ≤2). Residual and scatter plots indicated that the assumptions toward normality, linearity, and homoscedasticity were met.

Nine percent of observations at time 1 had missing values in the independent variables and were assessed with multivariate imputation by chained equations following the conditional multiple imputation approach (78). Associated factors from the regression analysis models were included in the imputation model for the dependent variable anxiety symptoms and for depressive symptoms, respectively. Twenty data sets were each imputed and subsequently pooled using Rubin's rules [(79); see (80) for an overview]. Sensitivity analyses were applied to explain any differences between the complete case analysis using list-wise deletion and the multiple imputation approach. All statistical analyses were conducted using R version 4.0.2. (81). *P*-values < .05 indicated statistical significance.

## Results

### Sample Characteristics

Participants at time 1 (*N* = 363) were mostly female (68%) and had a mean age of 26 years (*SD* = 4.27; Table 1). About half of the participants were single (49.2%), undergraduate students (46.4%), and the majority lived with others (75.2%). Most of the participants' parents (63%) had a middle economic status, while students themselves had an average income of 700–1000 Euro/month or less (67.1 %). On average, participants reported an overall very good or good health status (*M* = 1.76, *SD* = 0.73). Sixty-two participants (17.1%) self-reported a diagnosed mental disorder; most frequently named were anxiety and depressive disorders. Sociodemographic characteristics at time 1 did not differ between the cross-sectional sample and the longitudinal sample with matched data pairs, but slightly more participants with matched data had reported a mental disorder at time 1, χ^2^ (2, *N* = 498) = 9.63, *p* = .008 (Table 1).

**Table 1:** Sample characteristics.

	**Cross-sectional** **sample n (%)** **(*n* = 363)**	**Longitudinal** **sample n (%)** **(*n* = 135)**
**Age**		
mean (SD)	25.87 (4.69)	25.32 (3.83)
median	25	25
**Gender** ^**a**^		
female	247 (68.0%)	100 (74.1%)
male	116 (32.0%)	35 (25.9%)
**Family status**		
single	179 (49.2%)	68 (50.4%)
partnership	169 (46.6%)	61 (45.2%)
other	15 (4.1%)	6 (4.4%)
having children	19 (5.2%)	6 (4.4%)
**Living situation**		
with others	273 (75.2%)	96 (71.1%)
alone	90 (24.8%)	39 (28.9%)
**Highest degree**		
High school diploma	169 (46.4%)	71 (52.6%)
Bachelor's degree	158 (43.5%)	53 (39.3%)
Master's degree	36 (9.9%)	11 (8.1%)
**University in Berlin**		
Freie Universität	128 (35.3%)	57 (42.2%)
Technical University	55 (15.2%)	16 (11.9%)
Humboldt University	53 (14.6%)	18 (13.3%)
Other	127 (35.0%)	44 (32.6%)
**Field of study**		
Social sciences	146 (40.2%)	57 (42.2%)
Humanities and arts	73 (20.1%)	26 (19.1%)
Natural sciences	43 (11.8%)	16 (11.8%
Engineering	41 (11.3%)	9 (6.6%)
Economics and politics	40 (11.0%)	16 (11.8%)
Other	4 (1.1%)	2 (1.5%)
**Income (**€**)**		
<700	101 (27.9%)	38 (28.1%)
700-1000	142 (39.2%)	57 (42.2%)
1001–1300	60 (16.5%)	18 (13.3%)
>1301–1700	60 (16.5%)	22 (16.3%)
**Parents' SES**		
high	65 (18.6%)	22 (16.7%)
average	220 (63.0%)	87 (65.9%)
low	64 (18.3%)	23 (17.4%)
missing *n*	13 (3.6%)	3 (2.2%)
**Health status**		
mean *SD*)	1.76 (0.73)	1.77 (0.67)
missing *n*	21 (5.6%)	0 (0.0%)
**COVID risk group (yes)**	29 (8.5 %)	11 (8.1 %)
missing *n*	21 (5.6%)	0 (0.0%)
**Reported mental disorder (yes)**	62 (17.1%)	31 (23.0%)
missing *n*	21 (5.6%)	0 (0.0%)

*SD, standard deviation; SES, socioeconomic status.*

a *None of the participants identified as diverse*.

### Pandemic-Related Responses and Changes

Wellbeing since the pandemic was overall perceived as moderate (40%) or somewhat good (27.5%) at time 1, and slightly worse or worse (61%) compared to pre-pandemic levels (Table 2). Students stated to cope rather well (38.3%) with their daily lives since the pandemic and that their income at time 1 did not change (52.5%) compared to pre-pandemic levels. At time 1, students slightly agreed or agreed (43.5%) to fear a potential future lockdown, and at the same time, indicated they would cope rather well a second lockdown (46.3%). Students coped moderately well (30.0%) with their academic life's at time 1, which significantly improved from time 1 to time 2. The average number of days during the last 2 weeks having social contacts and drinking alcohol decreased compared to pre-pandemic levels, and decreased further from time 1 to time 2, respectively. All other ratings did not differ between the two assessments (Table 2).

**Table 2:** COVID-19 related responses and changes during the pandemic.

**Variables**	**Cross-** **sectional** **sample**	**Longitudinal sample**			
		**Time 1**	**Time 2**	**Paired *t* test (134)**	***p* value**
	*Mean* (SD)				
Perceived Wellbeing since COVID ^a^	2.65 (0.99)	2.65 (0.96)	2.66 (0.98)	−0.06	0.950
Wellbeing since COVID *vs*. pre-COVID ^a^	3.50 (1.01)	3.58 (1.00)	3.40 (1.04)	1.32	0.188
Coping with changes in daily life since COVID ^a^	2.48 (0.90)	2.51 (0.93)	2.36 (0.99)	1.26	0.209
Coping with academic life since COVID ^a^	2.92 (1.21)	3.01 (1.14)	2.65 (1.17)	2.41	0.017
Changed income since COVID (time 1) ^a^	3.44 (0.81)	3.36 (0.76)	-		
Afraid towards potential future lockdown (time 1) ^a^	3.14 (1.27)	3.13 (1.34)	-		
Coping with potential future lockdown (time 1) ^a^	2.67 (1.01)	2.65 (1.04)	-		
Days/last 2 weeks with social contacts	5.26 (3.72)	4.79 (3.76)	3.17 (2.95)	3.90	<0.001
Days/last 2 weeks with social contacts *vs*. pre-COVID	2.19 (1.07)	2.04 (1.00)	1.61 (0.84)	3.99	<0.001
Days/last 2 weeks drinking alcohol	3.33 (3.14)	3.08 (3.02)	2.07 (2.29)	3.08	0.002
Days/last 2 weeks drinking alcohol *vs*. pre-COVID	2.93 (0.98)	2.87 (0.92)	2.60 (0.97)	2.42	0.017

a *Higher scores indicate poor or worsened outcomes using a five-point Likert scale*.

### Mental Health Levels and Trajectories During the Pandemic

Table 3 shows the results for the primary mental health outcomes depressive symptoms and anxiety symptoms for the cross-sectional and the longitudinal sample. Prevalence rates in the cross-sectional sample were 38% and 26.5% for moderate-to-severe depressive and anxiety symptoms, respectively. In the longitudinal sample, prevalence rates did not change for depressive symptoms (43% vs. 43%) and anxiety symptoms (29.6% vs. 28.1%). Between time 1 and time 2, mean scores did not significantly differ for symptoms of depression, *t*(134) = −0.09, *p* = 0.924, *d* = −0.01, 95% CI [–0.16, 0.14], and anxiety, *t*(134) = −0.04, *p* = 0.968, *d* = −0.004, 95% CI [−0.17, 0.16]. Most participants showed asymptomatic PHQ-8 trajectories from time 1 to time 2 (44.4%), followed by stable symptomatic (30.4%), worsened (12.6%), and improved depressive trajectories (12.6%). Similarly, most GAD-7 trajectories during the pandemic were asymptomatic (56.3%), followed by improved (15.5%), stable symptomatic (14.1%), or worsened trajectories (14.1%).

**Table 3:** Primary outcomes and additional psychological variables during the COVID-19 pandemic.

**Variables**	**Cross-sectional sample**	**Longitudinal sample**					
		**Time 1**	**Time 2**	**Paired *t* test (134)**	***p***	**Cohen's *d***	**95 % CI**
Depression (PHQ-8)							
Mean (*SD*)	8.12 (5.24)	8.37 (5.52)	8.43 (4.63)	−0.09	0.924	−0.01	[−0.16, 0.14]
Median (*range*)	7.00 (0–23)	8.00 (0–23)	8.00 (0–21)				
Moderate-to-severe (*N*, %)	141 (38.8%)	58 (43.0%)	58 (43.0%)				
Anxiety (GAD-7)							
Mean (*SD*)	7.15 (4.64)	7.50 (4.71)	7.52 (4.30)	−0.04	0.968	−0.004	[−0.17, 0.16]
Median (range)	6.00 (0–21)	7.00 (0–21)	7.00 (0–20)				
Moderate-to-severe (*N*, %)	93 (25.6%)	40 (29.6%)	38 (28.1%)				
Loneliness							
Mean (*SD*)	15.61 (5.51)	15.96 (5.93)	17.61 (5.04)	−2.63	0.009	−0.30	[−0.47, −0.13]
Median (range)	14 (8–30)	14 (8–29)	17 (8–28)				
Stress ^a^							
Mean (*SD*)	3.91 (1.67)	4.09 (1.78)	4.42 (1.59)	−1.73	0.086	−0.20	[-0.37, −0.02]
Median (range)	4 (2–8)	4 (2–8)	4 (2–8)				
Satisfaction with life							
Mean (*SD*)	23.66 (6.58)	23.82 (6.59)	23.33 (6.17)	0.66	0.509	0.08	[−0.05, −0.21]
Median (range)	25 (5–34)	25 (6–34)	25 (5–34)				
Perceived social support							
Mean (*SD*)	19.83 (3.96)	19.96 (3.81)	19.98 (3.54)	−0.03	0.972	−0.004	[−0.14, −0.12]
Median (range)	21 (6–24)	21 (7–24)	21 (9–24)				
Repetitive negative thinking							
Mean (*SD*)	29.00 (12.59)	30.12 (12.67)	28.58 (13.10)	1.07	0.285	0.11	−0.03, −0.26]
Median (range)	29 (0–56)	30 (5–57)	29 (0–56)				

*SD, standard deviation; PHQ-8, patient health questionnaire-8; GAD-7, generalized anxiety disorder-7.*

a *Stress was measured with two items from the perceived stress scale (Cronbachs' α = 0.85)*.

Regarding the additional psychological outcomes, symptoms of stress and repetitive negative thinking did not significantly differ between time 1 and time 2 (Table 3). In addition, perceived social support and quality of life did not differ during the course of the pandemic. However, feelings of loneliness significantly increased among students between time 1 and time 2, *t*(134) = −2.63, *p* = 0.009. The effect size for increased loneliness was small, Cohen's *d* = −0.30, 95 % CI [−0.47, −0.13]).

**Figure 1:**
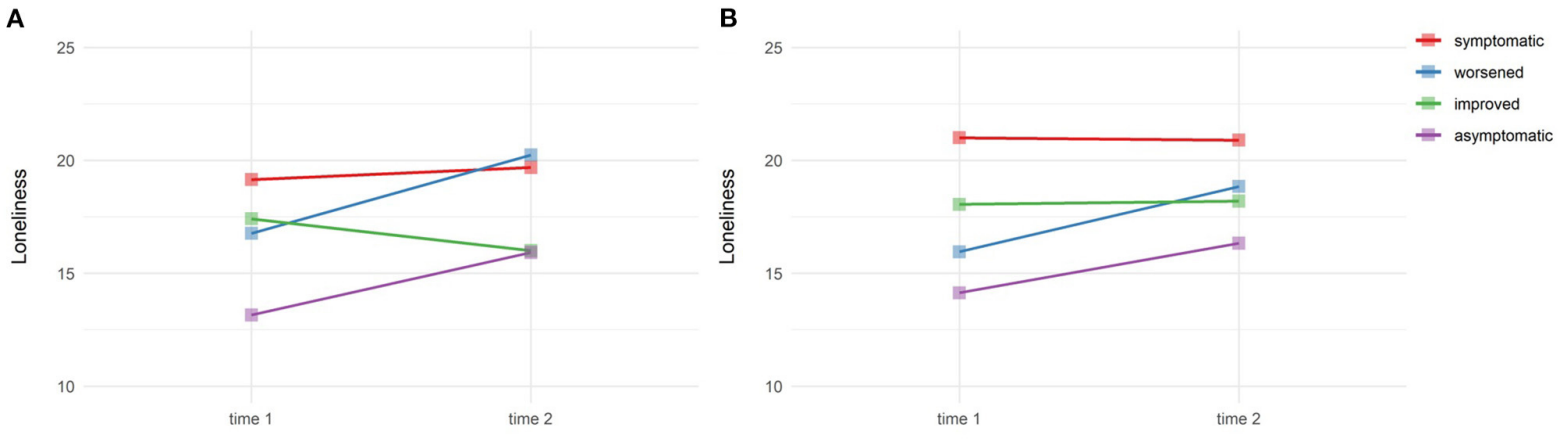
Feelings of loneliness and trajectories of depressive symptoms **(A)** and anxiety symptoms **(B)** during the COVID-19 pandemic.

To further explore the increases in loneliness during the pandemic, two-way repeated-measures ANOVAs were performed with loneliness across time and between different PHQ-8 and GAD-7 trajectories. Results indicated a large and significant difference in loneliness between the PHQ-8 trajectory groups, *F*(3, 131) = 11.49, *p* < 0.001, $\eta_{p}^{2}$ = 0.16, and a medium effect of time, *F*(1, 131) = 13.24, *p* < 0.001, $\eta_{p}^{2}$ = 0.03 (Figure 1). In addition, there was a significant and medium interaction effect between increased loneliness during the pandemic and the PHQ-8 trajectory groups *F*(3, 131) = 4.09, *p* = 0.008, $\eta_{p}^{2}$ = 0.02. Post hoc comparisons revealed that between time 1 and time 2, loneliness significantly increased in the asymptomatic PHQ-8 trajectory group (*p* = 0.002), while increases in the symptomatic and worsened courses did not reach statistical significance. At time 2, compared to asymptomatic PHQ-8 courses loneliness was more pronounced in groups with symptomatic (*p* = 0.007) and worsened courses (*p* = 0.043), but did not differ from the improved trajectory group (*p* = 0.998). Regarding GAD-7 trajectories, effects of group and time were statistically significant and large or medium-sized, *F*(3, 131) = 9.72, *p* < 0.001, $\eta_{p}^{2}$ = 0.13; *F*(3, 131) = 12.61, *p* < 0.001, $\eta_{p\;}^{2}=$ 0.02 (Figure 1). The overall interaction between GAD-7 trajectories and loneliness was not significant (*p* = 0.149, $\eta_{p}^{2}$ = 0.01). However, loneliness significantly increased in the asymptomatic GAD-7 trajectory group (*p* = 0.012). At time 2, loneliness was more prevalent in groups with stable symptomatic compared to asymptomatic GAD-7 courses (*p* = 0.014) and did not differ from worsened or improved GAD-7 trajectories (*p* = 0.548; *p* = 0.823).

### Associated Factors for Mental Health Levels and Changes

Table 4 shows the unadjusted and adjusted associations at time 1 with sociodemographic, pandemic-related and psychological variables, separately for the primary outcome of mental health (depressive symptoms, anxiety symptoms). When adjusting for all other tested variables, increased depressive symptoms at time 1 were associated with worse coping abilities in daily life (*B* = 0.64; *SE* = 0.29) and worse coping with academic life since the pandemic (*B* = 0.74; *SE* = 0.20), with higher levels of loneliness (*B* = 0.24; *SE* = 0.05), social anxiety (*B* = 0.20; *SE* = 0.08), boredom (*B* = 0.46; *SE* = 0.19), and repetitive negative thinking (*B* = 0.11; *SE* = 0.02; _*adj*_*R*^2^ = 52.3%, *p* < .001). Regarding anxiety symptoms at time 1, adjusted associations were found for living alone (*B* = −0.91; *SE* = 0.44), worse coping with academic life (*B* = 0.48; *SE* = 0.18), worse coping with a potential future lockdown (*B* = 0.51; *SE* = 0.26), loneliness (*B* = 0.20; *SE* = 0.05), and repetitive negative thinking (*B* = 0.13; *SE* = 0.02; _*adj*_*R*^2^ = 59.38%, *p* < .001 including all variables). Sensitivity analyses with complete data applying list-wise deleted revealed similar conclusions compared to the multiple imputation approach presented for both depressive and anxiety symptoms (see Supplementary Table 1).

**Table 4:** Associations with the two dependent variables depressive symptoms and anxiety symptoms at time 1 (N = 363) using simple linear (unadjusted models) and multiple linear regression analyses (adjusted models.).

	**Depressive Symptoms (time 1)**						**Anxiety Symptoms (time 1)**					
	**Unadjusted model**			**Adjusted model** ^**a**^			**Unadjusted model**			**Adjusted model** ^**a**^		
**Independent variables**	**Beta**	***SE***	***p***	**Beta**	***SE***	***p***	**Beta**	***SE***	***p***	**Beta**	***SE***	***p***
*Sociodemographic data (time 1)*												
Age	−0.08	0.06	0.157	−0.03	0.05	0.568	0.01	0.05	0.918	0.05	0.04	0.279
Gender (male vs.)												
female	1.15	0.58	0.052	0.14	0.44	0.743	1.32	0.52	0.011	0.29	0.40	0.463
Living situation (alone vs.)												
with others	0.45	0.64	0.478	−0.36	0.48	0.460	−0.48	0.56	0.391	−0.91	0.44	0.038
Family status (single vs.)												
partnership other	−0.80 −1.34	0.56 1.41	0.158 0.341	−0.32 0.32	0.44 1.05	0.472 0.763	0.08 −1.85	0.50 1.25	0.877 0.138	0.15 −0.11	0.40 0.96	0.705 0.909
Parents' SES (low vs.)												
middle high status	0.65 0.06	0.74 0.92	0.385 0.949	0.07 0.67	0.55 0.66	0.897 0.315	0.08 −0.70	0.66 0.83	0.121 0.396	−0.06 0.31	0.51 0.65	0.907 0.641
*Pandemic-related variables (time 1)*											
Students' income change	0.95	0.34	0.006	0.25	0.25	0.321	0.90	0.30	0.003	0.26	0.23	0.250
Coping with daily life	2.51	0.28	<0.001	0.64	0.29	0.029	2.16	0.25	<0.001	0.37	0.27	0.162
Coping with academic life	1.63	0.21	<0.001	0.74	0.20	<0.001	1.38	0.19	<0.001	0.48	0.18	0.007
Social contacts	−0.30	0.07	<0.001	−0.06	0.06	0.305	−0.30	0.06	<0.001	−0.08	0.06	0.149
Drinking alcohol	−0.06	0.09	0.515	0.07	0.08	0.341	−0.14	0.08	0.069	0.03	0.07	0.713
Coping future lockdown	1.37	0.27	<0.001	−0.05	0.28	0.863	1.56	0.23	<0.001	0.51	0.26	0.048
Anxiety future lockdown	−0.51	0.22	<0.001	0.19	0.20	0.320	−0.65	0.19	0.001	0.07	0.17	0.676
*Psychological variables (time 1)*												
Loneliness	0.53	0.42	<0.001	0.24	0.05	<0.001	0.45	0.38	<0.001	0.20	0.05	<0.001
Cope (positive reframing)	−1.07	0.17	<0.001	−0.18	0.15	0.254	−0.86	0.15	<0.001	−0.01	0.14	0.952
Cope (acceptance)	−0.69	0.17	<0.001	0.07	0.14	0.613	−0.81	0.15	<0.001	−0.24	0.13	0.061
Cope (substance use)	0.67	0.17	<0.001	0.22	0.14	0.128	0.23	0.15	0.136	−0.07	0.14	0.626
Social support	−0.48	0.07	<0.001	0.04	0.07	0.538	−0.42	0.06	<0.001	0.00	0.06	0.962
Self-efficacy	−0.44	0.56	<0.001	−0.03	0.05	0.579	−0.35	0.05	<0.001	0.04	0.05	0.451
Social anxiety	0.79	0.92	<0.001	0.20	0.08	0.016	0.58	0.08	<0.001	0.12	0.08	0.125
Boredom	1.77	0.23	<0.001	0.46	0.19	0.016	1.01	0.20	<0.001	−0.06	0.17	0.734
Repetitive negative thinking	0.24	0.19	<0.001	0.11	0.02	<0.001	0.21	0.02	<0.001	0.13	0.02	<0.001
Adverse childhood experiences	0.72	0.15	<0.001	0.19	0.12	0.109	0.54	0.13	<0.001	0.16	0.11	0.147
Current mental disorder (yes)	3.62	0.72	<0.001	0.80	0.56	0.154	2.82	0.65	<0.001	0.57	0.50	0.257
*R^2^* *adjusted R^2^*				0.556 0.523		<0.001 <0.001				0.528 0.493		<0.001 <0.001

*SES, socioeconomic status. Positive Beta values indicate a higher risk for depressive and anxiety symptoms.*

a *Adjusted for all other variables listed in the table*.

## Discussion

This study investigated the mental health levels and trajectories of university students during two different stages of the COVID-19 pandemic, i.e., at time 1 during the eased lockdown phase and at time 2 during the second lockdown in Germany. Contrary to the hypotheses, mean symptoms of depression and anxiety did not significantly change during the pandemic. Students most often had asymptomatic or sustained symptomatic courses of depression (56.3%, 30.4%) and anxiety (44.4%, 10.5%) during the pandemic; fewer worsened or improved between time 1 and time 2. Likewise, mean levels of stress, perceived quality of life, and social support did not change over the course of the pandemic. However, in line with the hypotheses, feelings of loneliness increased from time 1 to time 2. Higher levels of loneliness during the lockdown phase were present in participants with sustained or worsened symptom trajectories, while increases in loneliness were most prevalent in those with asymptomatic courses of depression and anxiety. Moreover, loneliness and repetitive negative thinking were associated with anxiety and depressive symptoms measured at time 1. Here, we discuss the results on mental health in the context of the COVID pandemic and its preventive measures (e.g., social distancing, closure of universities) together with further implications for students in higher education.

### Findings in Context

During the eased lockdown phase in July 2020 at time 1, anxiety (GAD-7) and depression (PHQ-8) scores were more than twice as high as normative and pre-pandemic data for German university students (9, 57, 82). However, mean scores and clinically relevant rates for depressive symptoms (38.8%) in our cross-sectional sample are comparable to pandemic data of German university students assessed between June and August 2020 [37%, (13); 38.5%, 29]. Prevalence rates for anxiety symptoms at time 1 were slightly lower as reported previously during the eased lockdown phase in Germany [25.6% vs. 35.5%; (29)]. Although rates for anxiety and depressive symptoms at time 1 were slightly higher in participants with matched data at time 1 and time 2 compared to the cross-sectional sample, our results fit in with the pooled prevalence rates of anxiety and depression among students from Western countries during the pandemic [e.g., (83, 84)].

In this study, anxiety and depressive symptoms did not change during the course of the pandemic and lockdown, which contradicts our hypotheses as well as recent findings among French students with increased rates between the eased lockdown phase and the second lockdown (40). However, symptom trajectories differed during the pandemic. While most students had asymptomatic courses, approximately four of ten students had a stable symptomatic or worsened depressive course, and three out of ten faced adverse anxiety courses during the pandemic. In addition, and contrary to our hypotheses, levels did not change regarding stress, quality of life, perceived social support, and repetitive negative thinking during the pandemic, reflecting previous mixed longitudinal data [e.g., (22, 36–38)]. First, our findings indicate that most university students reported slightly worse or worse wellbeing at time 1 compared with pre-pandemic levels, which is in line with prior research [e.g., (12, 27, 28, 31). These findings generally point to decreased wellbeing during the pandemic, while symptom levels likely persisted between time 1 and time 2. However, coping with academic life improved during the pandemic, which implies that most students adapted to the remote studying formats. Second, this study was conducted at the end of the semester at time 1, and elevated symptoms levels at time 1 may also have developed partly in response to the examination phase (32). The survey at time 2 was conducted shortly after the second lockdown had started, and its long-term consequences were possibly not yet tangible.

Consistent with previous assumptions and data (4, 12, 53) but inconsistent with others (31), loneliness increased among university students during the pandemic. Loneliness particularly increased in students with asymptomatic trajectories during the pandemic, and the highest levels were present in students with symptomatic or worse trajectories. This mirrors both trajectories and symptom levels during the pandemic among the general population compared to people with pre-existing mental disorders (51). Although aligned with the established social distancing measures, social contacts decreased compared with pre-pandemic levels and further decreased during the pandemic, likely fostering loneliness in the current sample. This finding raises concerns given that loneliness is a crucial risk factor for mental health in general (48) as well as a major reason for increased helpline calls during the pandemic in Germany, and worldwide (85).

Moreover, loneliness was significantly associated with both depressive and anxiety symptoms at time 1 along with repetitive negative thinking, while adaptive coping in daily and academic life was protective for depressive symptoms, and adaptive coping with a potential future lockdown was protective for anxiety symptoms. In addition, these findings generally fit within the literature (41–47), suggesting that the way students appraise the pandemic, as well as their connections with others, may be critical in understanding the mental health during the pandemic.

The current study provides novel evidence on mental health before and during a second lockdown, identified loneliness and repetitive negative thinking as salient risk factors for mental health, and demonstrated diverging trajectories of mental health in a homogenous sample of German university students. The findings on prevalent anxiety and depressive symptoms and increased loneliness during the pandemic may foster immediate preventive actions such as psychoeducation in higher education, but also stimulate research on interventions targeting loneliness among young adults. However, the study also faces limitations. First and most importantly, the sample sizes were small and the response rate at time 2 was low (43.3 %), increasing the risk for inflated data. These numbers are comparable to other studies on university students during the pandemic [e.g., (29)], but results should be replicated with representative and larger sample sizes. Our sample consisted of participants studying at Berlin-based universities, and thus our data may not be generalized to other (student) populations. Second, the current study assessed the mental health levels and changes only twice during the pandemic, as well as the pre-pandemic well-being in a retrospective manner. In the absence of pre-pandemic data, the consequences of the COVID-19 pandemic in this sample should be interpreted with caution. Third, all measures were self-administered via online surveys, which potentially confounds the validity of the results. We used cut-offs from the PHQ-8 and the GAD-7 to create subgroups with differential symptom trajectories (e.g., to study transitions from uncritical to clinically relevant states), which showed good sensitivity and specificity (58, 60). However, these measures cannot replace a structural clinical interview to diagnose a depression or anxiety disorder.

## Conclusion

Symptoms of anxiety and depression overall persisted during the COVID-19 pandemic while trajectories varied and feelings of loneliness significantly increased. Moreover, loneliness was associated with anxiety and depressive symptoms, indicating that preventing loneliness may help to maintain and promote mental health among university students. Representative studies on mental health, loneliness, and other associated factors are needed to fully identify students at high risk. Given that the COVID-19 pandemic and its preventive measures such as social distancing and remote learning continue for an indefinite period, long-term consequences for mental health are likely to occur, and universities should offer adequate support informed by the evidence to mitigate mental health problems and loneliness among university students.

## Data Availability Statement

The raw data supporting the conclusions of this article will be made available by the authors, without undue reservation.

## Ethics Statement

The studies involving human participants were reviewed and approved by Ethics Committee of the Freie Universität Berlin, Department of Education and Psychology. The participants provided their electronic informed consent to participate in this study.

## Author Contributions

MW, TB, and BR initiated this project and all authors contributed to designing and implementing the survey. MW analyzed the data and wrote the initial draft. All authors have reviewed and edited earlier versions and approved the final version of this manuscript.

## Conflict of Interest

The authors declare that the research was conducted in the absence of any commercial or financial relationships that could be construed as a potential conflict of interest.

## Publisher's Note

All claims expressed in this article are solely those of the authors and do not necessarily represent those of their affiliated organizations, or those of the publisher, the editors and the reviewers. Any product that may be evaluated in this article, or claim that may be made by its manufacturer, is not guaranteed or endorsed by the publisher.
