# Supplementary material for: Mental Health and Loneliness in University Students During the COVID-19 Pandemic in Germany: A Longitudinal Study
Source: Front Psychiatry. 2022 Apr 15;13:848645. doi: 10.3389/fpsyt.2022.848645 (PMC9051079; doi:10.3389/fpsyt.2022.848645)
Supplement: Supplementary file 1 [file Table_1.pdf]

SUPPLEMENTARY TABLE 1 | Associations with the two dependent variables depressive symptoms and anxiety symptoms at time 1 using simple linear (unadjusted models) and multiple linear regression analyses (adjusted models) with complete data.

	**Depressive Symptoms (time 1)**						**Anxiety Symptoms (time 1)**					
	**Unadjusted model**			**Adjusted model [a]**			**Unadjusted model**			**Adjusted model [a]**		
**Independent variables**	**Beta**	***SE***	***p***	**Beta**	***SE***	***p***	**Beta**	***SE***	***p***	**Beta**	***SE***	***p***
Sociodemographic data (time 1)												
Age	-0.07	0.06	.250	-0.02	0.4	.634	0.01	0.05	.876	0.05	0.04	.244
Gender (male vs.)												
female	1.07	0.63	.089	0.11	0.46	.814	1.27	0.54	.014	0.29	0.41	.479
Living situation (alone vs.)												
with others	-0.27	0.67	.686	-0.50	0.51	.326	0.67	0.58	.251	1.06	0.41	.018
Family status (single vs.)												
partnership	-0.46	0.60	.444	-0.20	0.46	.667	0.39	0.51	.452	0.28	0.41	.500
other	-1.11	1.48	.454	0.77	1.09	.481	-1.75	1.28	.173	-0.10	0.97	.921
Parents' SES (low vs.)												
middle	0.22	0.77	.772	-0.01	0.57	.979	-0.10	0.67	.879	0.09	0.45	.853
high status	-0.23	0.95	.807	0.58	0.69	.406	-0.73	0.83	.830	0.47	0.61	.440
Pandemic-related variables (time 1)												
Students' income change	1.10	0.36	.003	0.36	0.26	.178	1.01	0.31	.027	0.37	0.23	0.11
Coping with daily life	2.62	0.29	<.001	0.75	0.31	.017	2.10	0.26	<.001	0.17	0.27	.538
Coping with academic life	1.69	0.22	<.001	0.74	0.21	<.001	1.41	0.19	<.001	0.46	0.23	.012
Social contacts	-0.31	0.77	<.001	-0.09	0.07	.154	-0.28	0.07	<.001	-0.08	0.06	.178
Drinking alcohol	-0.07	0.09	.457	0.08	0.08	.332	-0.16	0.08	.053	0.01	0.07	.881
Coping future lockdown	1.35	0.28	<.001	-0.05	0.29	.869	1.63	0.24	<.001	0.67	0.26	.009
Anxiety future lockdown	0.58	0.22	.016	0.14	0.20	.486	0.79	0.20	<.001	-0.03	0.18	.879

SUPPLEMENTARY TABLE 1 | Continued

Psychological variables (time 1)												
Loneliness	0.54	0.44	<.001	0.22	0.54	<.001	0.47	0.39	<.001	0.22	0.04	<.001
Cope (positive reframing)	-1.06	0.18	<.001	-0.14	0.16	.398	-0.87	0.16	<.001	-0.03	0.14	.979
Cope (acceptance)	-0.59	0.18	<.001	0.10	0.14	.487	-0.74	0.15	<.001	-0.16	0.13	.197
Cope (substance use)	0.72	0.18	<.001	0.25	0.15	.102	0.29	0.16	.067	0.01	0.13	.989
Social support	-0.5	0.07	<.001	0.01	0.07	.848	-0.42	0.06	<.001	-0.01	0.06	.908
Self-efficacy	-0.44	0.56	<.001	0.00	0.06	.989	-0.36	0.05	<.001	0.05	0.05	.353
Social anxiety	0.79	0.92	<.001	0.22	0.09	.011	0.59	0.08	<.001	0.12	0.08	.125
Boredom	1.77	0.23	<.001	0.53	0.20	.008	1.11	0.21	<.001	-0.02	0.17	.907
Repetitive negative thinking	0.24	0.19	<.001	0.11	0.02	<.001	0.21	0.02	<.001	0.13	0.02	<.001
Adverse childhood experiences	0.72	0.15	<.001	0.18	0.12	.141	0.54	0.13	<.001	0.11	0.11	.288
Current mental disorder (yes)	3.47	0.74	<.001	0.90	0.59	.128	2.80	0.65	<.001	0.80	0.52	.129
R^2					0.562	<.001					.546	<.001
Adjusted R^2					0.526	<.001					.509	<.001

SES = socioeconomic status. 34 from 363 observations deleted due to missingness. Positive Beta values indicate a higher risk for depressive and anxiety symptoms.

[a] *Adjusted for all other variables listed in the table.*
